# Study of the noise spectrum on high frequency thresholds in workers exposed to noise

**DOI:** 10.1590/S1808-86942012000400020

**Published:** 2015-10-20

**Authors:** Aurea Oliveira Canha Ottoni, Anadergh Barbosa-Branco, Marlene Escher Boger, Sérgio Luiz Garavelli

**Affiliations:** 1 PhD in Health Sciences - UnB (Speech and Hearing Therapist); 2 PhD in Occupational Health (Professor - University of Brasília - UnB); 3 MSc in Health Sciences (Speech and Hearing Therapist); 4 PhD in Physics (Professor - Catholic University of Brasília - UCB)

**Keywords:** audiometry, hearing loss noise-induced, noise measurement, spectrum analysis

## Abstract

Noise level can be quantified and qualified based on sound characteristics such as intensity, type of spectrum, duration and distribution of the noise exposure during one's working hours.

**Objective**: To assess noise spectrum and the audiometric configuration of workers.

**Materials and Methods**: Contemporary cross-sectional cohort carried out in the Federal District Brazil. We did an environmental analysis (spectral analysis) of the noise in companies from different industries, with audiological assessment of 347 workers.

**Results**: The spectral analysis revealed peaks at different frequencies for each industry investigated (8 kHz-metallurgical, 4 kHz-stone Works and 2 kHz-wood works). We noticed that the frequencies of 14 kHz and 16 kHz had significant differences between the various industries, with a greater prevalence of the metallurgical.

**Conclusion**: The use of noise pressure measuring device, coupled to a frequency analyzer and high frequency audiometric assessment yielded an early detection of hearing damage, helping better organize preventive measures.

## INTRODUCTION

The noise level of harm may be quantified and qualified based on some sound characteristics, such as its intensity, the type of spectrum, duration and noise exposure distribution throughout the day of work^1^.

These measures can be obtained using sound pressure measuring devices coupled to frequency analyzers. Such devices show the sound pressure level (SPL) corresponding to the selected frequency band (sound spectrum). Thus, environment noise monitoring helps in the acoustic classification of operational units, enabling the identification of the noisiest places and equipment, collecting information which will be used to select and optimize individual hearing protection devices^2^, ^3^.

Audiometric evaluation is the most used procedure in environments with noise above 85 dB(A). Such assessment aims at protection the worker's hearing and is fundamental in Hearing Protection Programs - HPP^4^, ^5^, ^6^, ^7^, ^8^, ^9^, ^10^, ^11^, ^12^, ^13^, ^14^, ^15^, ^16^, ^17^, ^18^, ^19^, ^20^. Conventional audiometry is but a graphic recording of hearing thresholds at different sound frequencies, which vary between 0.125 kHz and 8 kHz^21^, ^22^, ^23^, ^24^, ^25^, ^26^, ^27^, ^28^, ^29^, ^30^, ^31^, ^32^, ^33^, ^34^, ^35^, ^36^, ^37^, ^38^. However, with the evolution of occupational audiology, recent studies have suggested the use of high frequency audiometry (above 8 kHz) as a clinical resource which adds greater sensitivity to such assessment, and provides for the early detection of cochlear problems^4^, ^5^.

Upon comparing the results obtained in conventional audiometry with those from high frequency audiometry in workers exposed and in those not exposed to noise, a study showed that the tonal thresholds were significantly higher in the high frequencies (*p* < 0.05) in the group with noise exposure, concluding that such approach enabled the early detection of hearing loss among the workers^6^.

Despite numerous recent studies assessing the effects of noise in worker's health^2^, ^7^, ^8^, ^9^, ^10^, ^11^, ^12^, ^13^, ^14^, ^15^, ^16^, ^17^, ^18^, ^19^, ^20^, ^21^, ^22^, ^23^, ^24^, ^25^, ^26^, ^27^, ^28^, ^29^, ^30^, ^31^, ^32^, ^33^, ^34^, ^35^, ^36^, ^37^, ^38^, studies utilizing the combination of environmental with biological monitoring are still rare. We should explore more the analysis of the frequency spectrum in the work environment. Studying the sound pressure levels breakdown into frequency bands, it is possible to obtain more information about the noise, providing better guidance for the worker's hearing protection program. Considering such *gap*, the goal of the present study was to assess the influence of the sound spectrum in the hearing thresholds in the high frequencies in workers from different industries in the Federal District of Brazil.

## MATERIALS AND METHODS

This is a contemporary study with a cross-sectional cohort, carried out in the city of Brasília, FD, between January of 2008 and December of 2010.

The criteria utilized to select the industries were: acceptance to participate in the study; be located in the Federal District; have, mainly, work environments with noise levels higher than 85 dB(A); find in the national classification of economic activities as risk grade 3 or 4, according to the NR 4, Charts I and II of the Ministry of Labor^10^. The companies who met the aforementioned criteria were grouped according to the type of activity, resulting in four different industries: stone works, wood works, metal works and cement manufacturing. Four stone works (62.5%), two wood works (25.0%), one metal works (6.2%) and one cement manufacturer (6.2%), adding up to a total of 347 workers, all males.

Among the workers, we selected those who met he following inclusion criteria: work for at least one year in the current position in an 8-hour shift; not having worked in another activity with noise exposure; not working directly with chemicals; not using ototoxic drugs, not having a history of high frequency acoustic trauma, be a male, be in an age range between19 and 65 years; accept to participate in the study and sign the informed consent form.

### Environment Assessment

Checking the sound pressure levels, dose and spectrum were made by a team specialized in environment acoustics. As far as sound pressure levels (SPL) and frequency spectrum are concerned, the measurements were carried out in a central point of the facility, capturing the general noise of the environment. In order to check the SPL, we used the sound pressure level measuring device coupled to the frequency analyzer model SIP-95, manufactured by 01 dB Brazil. This equipment was properly calibrated by a specialized company, with frequency filters in bands of 1/3 and 1 of octave, operating in an “A” slow response compensation circuit (*SLOW)*, as per established by Ordinance # 19, from 04/09/98, of the Ministry of Labor and Employment^11^. In order to carry out dosimetry measures, we used a digital, compact, wireless device, SOLO (*SIE Badge)* model from 01 dB - Brazil. For assessment purposes, we selected one worker who worked in the noisiest machine of each industry. The reference criterion which serves as foundation for the daily exposure limits adopted for a continuous or intermittent noise corresponds to a dose of 100% for the exposure of eight hours at the level of 85 dB(A)^12^. Eight hours were considered as the mean time of daily exposure (T_exp_) for a work shift (T_0_). The equivalent noise level - L_eq_ - represents the equivalent level of sound pressure, according to the definition of the NBR (10.151)^13^: the evaluation criterion considers, besides the reference criterion, the increment in dose duplication (q) equal to 5 and L_crit_ of 85 dB(A)^14^. In order to check if the environment followed the regulatory norms associated with noise, we chose to calculate the equivalent level of sound pressure (L_eq_), which represents a continuous noise level in dB(A).

### Audiological Assessment

The audiometric assessment was made up by conventional audiometry (0.25 kHz to 8 kHz), as per established by the Ministry of Work and Labor^11^, and by high frequency audiometry (9 kHz and 16 kHz), both carried out under hearing rest of 14 hours. These assessments were preceded by inspecting the external acoustic meatus of each worker, in order to rule out obstruction or other conditions that could impact the results of the assessment.

All the tests were carried out in the employer's facilities, under favorable conditions to the test (silent places).

In order to do the audiological assessment, we used the following devices: *Welch Allyn* otoscope with WA accessories; *Reduson* (portable) audiometry booth; two-channel clinical audiometer from *Interacoustic*, model AD 40 with a TDH 39 phone, in order to check the tonal threshold between 0.25 and 8 kHz. In order to assess the frequencies above 8 kHz, we used the high frequency *KOSS HIPRO phone*.

In conventional audiometry, the procedure was based on checking the minimum response level for air stimuli for the frequencies between 0.25 kHz and 8 kHz. The worker who had hearing thresholds above 25 dBHL in the frequencies of 0.5 kHz and 4 kHz was also submitted to checking hearing thresholds by bone conduction. The high frequency audiometry analyzed the audiometric thresholds in the frequencies of 9 kHz, 10 kHz, 12 kHz, 14 kHz and 16 kHz.

The results from occupational audiometry were grouped according to the criteria specified below in only two categories: normal audiometry - all audiometries which had tonal values equal to or lower than 25 dBHL in all the frequencies; and audiometry levels suggesting Noise Induced Hearing Loss (NIHL) - all audiometries which had a notch higher than 25 dBHL in at least one of the frequencies from 3 kHz. Thresholds higher than 25dBHL were considered a notch in one or more isolated frequencies, as a recovery in the following frequency^15^.

The database was created in *Excel*® format. The analysis was made using the SPSS 13® (*Statistical Package for the Social Sciences*, Chicago, IL) for *Windows®* and *SigmaStat* 3.11® for *Windows®*. The chi-squared test was utilized to check for possible associations between variables. We considered the results from each ear, subdivided into right and left ear and the audiometric data was inserted in a variance analysis model of mixed design with the factors: industry of the worker (four levels; independent measure) and frequency (13 levels; repeated measure). The multiple comparison procedure utilized the Bonferroni correction method. For the additional *pos hoc* analysis, we used the ANOVA test of one path. The statistical significance level was established as 5% (*p* < 0.05). All the tests were bicaudal.

This study was approved by the Ethics in Research Committee, recorded as 048/2004. All the workers who participated signed the informed consent form.

## RESULTS

### Assessment of the Environment

The environment assessment was carried out in three industries (wood works, stone works and metal works). Such measurements could not be checked in the cement factory, because the company did not authorize it.

Considering the equivalent level of sound pressure (L_eq_) as an indicator of environment noise spectrum in the different companies, the results are shown on Table 1. The L_eq_ value points to the noise intensity in the three industries and in the type of spectrum found: frequency peak at 8 kHz in the metallurgical industry, 4 kHz in stone works and 2 kHz in wood works. In the metallurgical plant, we noticed the highest L_eq_ value if compared to those from the other industries, with intensities of 85.4 dB (Table 1).

**Table 1:** Distribution of the sound pressure equivalent level (L_eq_) in octave bands.

Octave band (kHz)	Wood	Industries(1) L_eq_ (dB) Stone	Metal
OIT 31.5	62.4	64.2	71.5
OIT 63	60.1	63.8	71.7
OIT 125	65.8	63.1	76.6
OIT 250	72.2	66.6	82.6
OIT 500	73.9	71.0	81.8
OIT 1	78.6	73.2	81.2
OIT 2	80.5	74.9	81.0
OIT 4	73.7	79.3	82.2
OIT 8	71.0	71.7	85.4
OIT 16	58.7	62.0	77.9
Global L_eq_	84.3	82.5	91.0

(1) This indicator was not assessed in the cement industry.

Concerning the assessment of the individual dose of the sound pressure level (SPL) in the three industries that the values went beyond the tolerance thresholds defined as 100%. Among these industries, the wood had a dose which was 29 times higher than the legally established value^12^. In exposures to such dose, the tolerance time allowed to the worker without using ear protection is 18.4 minutes. We should stress that the workers assessed have a minimum work load of 8 hours a day. In relation to the general sound pressure level analysis, we noticed that the noise intensity level was higher than 100 dB SPL in the three industries and the wood works had the highest sound pressure levels (108.5 dB(A)) (Table 2).

**Table 2:** Sound pressure equivalent level (L_eq_), level of daily exposure (L_exp_), dose and maximum tolerated exposure time according to the industry - Federal District - 2010.

Industry	L_eq_ dB(A)	L_exp_ dB(A)exp	Dose (%)	Tolerated time (min)
Wood works	108.5	109.0	2924.1	18.4
Stone works	104.5	105.0	1679.4	32.1
Metal works	103.3	103.8	1422.0	37.9

L_eq_: sound pressure equivalent level; L_exp_: daily exposure level.

### Clinical Assessment

In the anamnesis, there were reports of clinical problems, such as difficulties to hear, otalgia, vertigo and tinnitus with the possibility of having more than one complaint per worker.

Data concerning the individual protection equipment (IPE) showed that 285 workers (82.1%) reported they used it. The workers in the wood works industry are the ones that least use hearing protection (42.6%). On the other hand, the workers of the metallurgical and stone works industries stood out in using IPE.

### Audiological Assessment

Table 3 shows the general characteristics of the audiometric results as to auditory thresholds. Notches were the thresholds above 25 dBHL in, at least, one frequency and one ear. Upon considering only the results from conventional audiometry, 55.3% of the workers assessed had some type of auditory notch suggestive of NIHL. Nonetheless, when the high frequencies were analyzed, the prevalence of auditory notch went up to 78.0% (Table 3).

**Table 3:** Distribution of the variables associated with the audiometric results (n = 694) in the four industries - Federal District - 2010.

Variable	n	%	% valid
Medical Report			
Normal hearing	108	15.6	-
Hearing loss	11	1.6	-
Notch	575	82.9	-
Conventional audiometry notch			
No frequency	203	29.3	34.6
1 frequency	122	17.6	20.8
2 to 3 frequencies	203	29.3	34.6
All the frequencies	58	8.4	9.9
Notch high frequency audiometry			
No frequency	45	6.5	7.7
1 frequency	149	21.5	25.4
2 to 4 frequencies	253	36.5	43.2
All frequencies	139	20.0	23.7
Alteration prevalence			
0.25 kHz	42	6.1	7.2
0.5 kHz	21	3.0	3.6
1 kHz	22	3.2	3.8
2 kHz	41	5.9	7.0
3 kHz	143	20.6	24.4
4 kHz	225	32.4	38.4
6 kHz	315	45.4	53.8
8 kHz	164	23.6	28.0
9 kHz	177	25.5	30.2
10 kHz	203	29.3	34.6
12 kHz	287	41.4	49.0
14 kHz	364	52.4	62.1
16 kHz	523	75.4	89.2

% valid in regards to the total number of subjects who had some change in the test (n = 586).

Among the frequencies with a notch, we found a higher sensitivity in the high frequency results (92.3%) when compared to the conventional frequencies (65.3%). Upon analyzing each frequency separately, we found a greater prevalence (75.4%) in the frequency of 16 kHz. Although we have found an increase in the prevalence of a notch suggestive of NIHL in the frequency of 6 kHz (53.8%), this data was not statistically significant (Table 3).

The analysis of the association between the industry and the auditory notch suggestive of NIHL showed a statistically significant association between the presence of the notch in the frequencies of 8 kHz, 12 kHz, 14 kHz and 16 kHz and the industry, with a weak power of association (CC*: 0.131, 0.141, 0.162 and 0.131, respectively).

In the results from the high frequency audiometries, we noticed a significant effect of the age co-variable (F_1, 689_ = 218,235, *p* < 0.001). The mean value of the results from workers with more than 40 years was significantly higher in comparison to workers below 40 years of age (*p* < 0.001). The effect of the industry co-variable was not significant on the mean values of the audiometric thresholds (F_3, 689_= 0.340, ***p*** = 0.796), in opposition to the sound frequency variable (F_4, 2756_ = 17.317, ***p*** < 0.001). The multiple comparisons procedure found significant differences among all the frequencies, except at 9 and 10 kHz (*p* = 0.318).

Concerning the interaction between the industry and the frequency, it was statistically significant (F_1, 2756_ = 2.645, *p* = 0.011). *Pos hoc* analyses showed significant differences between the industries on the frequencies of 14 kHz (F_3, 690_ = 3.747, *p* = 0.011) and 16 kHz (F_3, 690_ = 3.5 1 5, *p* = 0.015). The stone works had lower audiometric thresholds than the cement manufacturing (14 kHz, *p* = 0.006) and the metallurgy (14 KHz and 16 kHz, *p* = 0.003, *p* = 0.001, respectively).

Concerning the industry variable and the mean value of audiometric thresholds, we noticed that on frequencies 0.25 kHz to 16 kHz, regardless of industry, there was a similarity in the audiometric trace, and as it progresses to the high frequencies, the auditory threshold would increase. Although the 6 kHz, 9 kHz and 12 kHz frequencies had differences between the industries, only the 14 kHz and 16 kHz were statistically significant (*p* = 0.011, 0.015, respectively).

Comparing the mean audiometric thresholds and the results found in the spectral analysis of each industry assessed, we noticed that the metallurgical industry had the highest audiometric threshold mean values in the high frequencies when compared to the other industries, and it had higher global L_eq_ than the other industries (91.0 dB) (Table 4).

**Table 4:** Comparing the audiometric thresholds and the results from the spectral analysis, according to frequency octave bands and the industry - Federal District - 2010.

Octave band (Hz)	Industries								
	Wood			Stone			Metal		
	L_eq_ (dB)	Mean (dB)	SE	L_eq_ (dB)	Mean (dB)	SE	L_eq_ (dB)	Mean (dB)	SE
31.5	62.4	-	-	64.2	-	-	71.5	-	-
63	60.1	-	-	63.8	-	-	71.7	-	-
125	65.8	-	-	63.1	-	-	76.6	-	-
250	72.2	14.4	0.6	66.6	16.3	0.3	82.6	15.8	0.5
500	73.9	12.5	0.6	71.0	13.3	0.3	81.8	12.7	0.5
1	78.6	10.4	0.7	73.2	10.7	0.4	81.2	10.9	0.5
2	80.5	12.0	0.9	74.9	11.5	0.6	81.0	10.8	0.6
4	73.7	21.6	1.5	74.3	21.1	1.3	82.2	20.8	1.1
8	71	16.5	1.3	71.7	18.9	1.2	85.4	17.8	1.1
16	58.7	41.1	1.8	62.0	38.5	1.6	77.9	45.4	1.5
Global L	84.3	_-_	_-_	82.5	_-_	_-_	91	_-_	_-_

L_eq_ sound pressure equivalent level, mean value of audiometric thresholds. SE: Standard Error.

## DISCUSSION

This study tried to investigate the association between the noise spectrum and the audiometric configuration in workers from different industries exposed to occupational noise, and it was possible to identify factors associated with the work conditions, which can predispose such patients to hearing impairment.

The prevalence of NIHL found in this study corroborates most of the literature findings^2^, ^6^, ^8^, ^9^, ^16^, confirming world statistical data, which characterizes NIHL as a public health problem^14^, ^17^, ^18^, besides being the second most common form of sensorineural hearing loss, after presbycusis^7^.

We noticed that the mean value of audiometric thresholds and the presence of a NIHL-suggestive notch^6^, ^9^, ^20^ were significant in the high frequencies. In the present study, the prevalence of notches in the high frequencies follows that of other authors, who noticed a higher prevalence of hearing loss in the high frequencies in workers with a past of exposure to high noise levels^20^, ^21^, ^22^.

The fact that the prevalence of the NIHL-suggestive notch is greater in the high frequencies could be explained because of the very cochlear anatomy and function dynamics, besides the lack of vascularization in the cochlear basal region^15^. Some studies suggest that in the high frequencies there is greater auditory sensitivity with aging, when compared to the lower frequencies^23^, ^24^. Such findings corroborate the literature and indicate that high frequency audiometry could be clinically utilized for the early diagnosis of NIHL^20^, ^21^, ^22^.

In Brazil, NR 7 from the Ministry of Work and Labor^11^ defines that the audiometric test must be always made through the frequencies from 0.5 kHz to 8 kHz; however, there is still no standardization for high frequency audiometry. Nonetheless, the number of workers exposed to high levels of sound pressure demands the need to understand and evaluate the risk that such exposure brings to health. In cases of technical impossibility to do the high frequency audiometry, we suggest the evoked otoacoustic emissions test, which enables the assessment of the outer hair cells, which can be checked in the frequencies up to 10 kHz, besides being a fast and objective test^25^.

Thus, we can state that without doing the high frequency audiometry, it would not have been possible to detect the worker's hearing loss in an initial stage. Therefore, this procedure was fundamental for the early detection of the NIHL-suggestive notch; this fact has also been pointed out by other authors^2^, ^4^, ^6^, ^20^, ^22^. Having all of this, it is paramount to reassess the guidelines and parameters of the assessment required by law, whenever the goal is to promote and protect the worker's health.

The results from the conventional audiometric thresholds as to the high frequency audiometry, presented significant difference (*p* < 0.001) in the workers older than 40 years. It is worth stressing that the high level of sound pressure associated to the years of profession and/or to age may compromise not only the hearing, but also the well-being of the worker^27^.

Upon investigating the environmental condition of the different industries, with the aim of checking for possible associations with the type of audiometric configuration of the worker in each environment, it was noticed that the results from the audiological alterations did not coincide with the frequency spectrum found in each industry. Nonetheless, in the metallurgical setting, the noise spectrum found presented a spectral frequency peak of 8 kHz. It is known that this frequency (8 kHz) is located in a region near that of the highest cochlear vulnerability^2^, ^4^, ^8^.

In the assessments carried out among the different industries, we found a sound pressure level higher than 100 dB, with a daily dose higher than what is allowed by law (100%). Such result has corroborated the ones in the literature^7^, ^8^, ^28^, indicating that exposure to intense noise, without proper hearing protection is one of the NIHL–triggering factors^1^, ^29^. According to the ***American Academy*** *of Otorhinolaryngology - Head and Neck Surgery*^30^, there are four factors which can impair the worker's hearing: high sound pressure level (SPL), SPL spectral distribution, noise duration and distribution and cumulative exposure to noise in days, weeks or years.

In regards of the results found, two industries stood out: the metallurgical and wood works. Upon the environment assessment, the metallurgical had higher intensities than the others, with a global L_eq_ of 91.0 dB, high frequency noise spectrum (peak at 8 kHz), sound pressure level of 103.3 dB(A), and dose higher than 100% (1422.0%). The statistical analysis of the assessments carried out indicated a statistically significant association (*p* < 0.005) of having notches in the high frequencies (12 kHz, 14 kHz and 16 kHz). These findings draw our attention, because this industry stands out in IPE compliance. Thus, such findings bring about some questions: the use of an ear IPE would be inadequate, or the model would not meet the type of noise found. The physical characteristics of the noise produced in the metallurgical industry would be more harmful that what is seen in the other industries assessed, affecting the high frequencies in a greater proportion.

In the wood works, the workers were the ones submitted to higher sound pressures (108.5 dB(A)), with a higher dose of noise during their work hours (2,924.1%), and who had lower compliance towards using an ear IPE. Contrary to what was expected, these industries (cement factory, wood, stone and metal) were the ones which had significant lower data concerning the presence of NIHL–suggestive notch when compared to the other industries assessed (*p* < 0.031). This evidence may be partially justified by the fact that the spectrum frequency peak found in such industry was of 2 kHz, because this is a frequency which is not located on the cochlear base^4^, ^6^. Exposure to this noise must not cause so much auditory damage as it happens in companies in which the spectral frequency peak is not so high.

The high prevalence of a NIHL-suggestive notch tells us that, depending on the noise dominant frequency (sound spectrum), there is an extension of the auditory effects^30^, ^33^. One study carried out with metallurgical workers exposed to high sound pressure levels (higher than 85 dB(A) concluded that cochlear problems are more harmful when the noise is made up of high frequencies^2^.

It was not possible to establish a clear association between the audiological configuration and the noise spectrum in the different industries. Nonetheless, it is suggested that the type of noise spectrum may impact the audiometric results and, in order to prove such fact, it is necessary to have a study involving one sample extended to other industries, which spectral peak is in lower frequencies.

In the present study, the assessment of the spectral type was fundamental to understand the high prevalence of a NILH-suggestive notch in the metallurgical industry and in the creation of a hypothesis which would justify the audiometric results found in the wood industry. Therefore, we believe special attention must be given to the type of noise measured in the companies, because most of the times, when such checking happens, it is done by using only the sound pressure level measuring device and not the frequency analyzer.

It is not enough to legally have a hearing protection program. It is necessary to have proper proposals to change the work environment in a more guided way, as, per example, understanding of the type of noise spectrum and high frequency analysis. Moreover, the information given the workers about the use of an ear IPE^34^, ^35^ must be clear and the company must do a periodic individual follow up which avoids or prevents hearing losses^36^. Therefore, preventive measures emphasizing hearing monitoring, including analyses of the high frequencies^37^, ^38^, besides controlling and correcting environmental conditions of the company with frequency analyzers^28^ and constant follow up of the worker's health, are decisive stages in controlling the damage that noise can bring to the worker's health.

This study found some hurdles in its execution, among them we stress that the companies did not accept well to participate in this study, which positively contributed to less severe results than those potentially presented by the set of companies in that given industry. It is possible that the companies acceptance is associated to the idea of better work conditions. Another factor which hampered this study was the lack of authorization to carry out the spectral assessment and the dosimetry in the cement factory.

## CONCLUSION

We did not find significant associations concerning the audiometric configuration and the noise spectrum standard which would enable to check the influence of spectral profiles on hearing loss, recorded by audiometric responses of the workers assessed.
